# Gap Analysis Regarding Prognostication in Neurocritical Care: A Joint Statement from the German Neurocritical Care Society and the Neurocritical Care Society

**DOI:** 10.1007/s12028-019-00769-6

**Published:** 2019-07-31

**Authors:** Katja E. Wartenberg, David Y. Hwang, Karl Georg Haeusler, Susanne Muehlschlegel, Oliver W. Sakowitz, Dominik Madžar, Hajo M. Hamer, Alejandro A. Rabinstein, David M. Greer, J. Claude Hemphill, Juergen Meixensberger, Panayiotis N. Varelas

**Affiliations:** 1 Neurocritical Care and Stroke Unit, Department of Neurology, University of Leipzig, Liebigstrasse 20, 04103 Leipzig, Germany; 2 Department of Neurology, Yale School of Medicine, P.O. Box 208018, New Haven, CT 06520-8018 USA; 3 Department of Neurology, Universitätsklinikum Würzburg, Josef-Schneider-Strasse 11, 97080 Würzburg, Germany; 4 Department of Neurology, Anesthesiology and Surgery, University of Massachusetts Medical School, 55 Lake Avenue North, Worcester, MA 01655 USA; 5 Neurosurgery Center Ludwigsburg-Heilbronn, RKH Klinikum Ludwigsburg, Posilipostrasse 4, 71640 Ludwigsburg, Germany; 6 Department of Neurology, University of Erlangen, Schwabachanlage 6, 91054 Erlangen, Germany; 7 Department of Neurology, Mayo Clinic, 200 First St SW, Rochester, MN 55905 USA; 8 Department of Neurology, Boston University Medical Center, 72 East Concord St, Boston, MA 02118 USA; 9 Department of Neurology, University of California San Francisco, 1001 Potrero Ave, San Francisco, CA 94110 USA; 10 Department of Neurosurgery, University of Leipzig, Liebigstrasse 20, 04103 Leipzig, Germany; 11 Department of Neurology and Neurosurgery, Henry Ford Hospital, 2799 W. Grand Blvd Neurosurgery - K-11, Detroit, MI 48202 USA

**Keywords:** Prognostication, Self-fulfilling prophecy, Outcome predictors, Comorbidities

## Abstract

**Background/Objective:**

Prognostication is a routine part of the delivery of neurocritical care for most patients with acute neurocritical illnesses. Numerous prognostic models exist for many different conditions. However, there are concerns about significant gaps in knowledge regarding optimal methods of prognostication.

**Methods:**

As part of the Arbeitstagung NeuroIntensivMedizin meeting in February 2018 in Würzburg, Germany, a joint session on prognostication was held between the German NeuroIntensive Care Society and the Neurocritical Care Society. The purpose of this session was to provide presentations and open discussion regarding existing prognostic models for eight common neurocritical care conditions (aneurysmal subarachnoid hemorrhage, intracerebral hemorrhage, acute ischemic stroke, traumatic brain injury, traumatic spinal cord injury, status epilepticus, Guillain–Barré Syndrome, and global cerebral ischemia from cardiac arrest). The goal was to develop a qualitative gap analysis regarding prognostication that could help inform a future framework for clinical studies and guidelines.

**Results:**

Prognostic models exist for all of the conditions presented. However, there are significant gaps in prognostication in each condition. Furthermore, several themes emerged that crossed across several or all diseases presented. Specifically, the self-fulfilling prophecy, lack of accounting for medical comorbidities, and absence of integration of in-hospital care parameters were identified as major gaps in most prognostic models.

**Conclusions:**

Prognostication in neurocritical care is important, and current prognostic models are limited. This gap analysis provides a summary assessment of issues that could be addressed in future studies and evidence-based guidelines in order to improve the process of prognostication.

## Introduction

The word *prognostication* derives from the ancient Greek word πρόγνωσις (προ [pro-; before] + γνώσις [gnosis; knowledge] or γιγνώσκειν [verb; come to know). Prognostication involves an attempt to predict the future or, specifically in medicine, the course of a disease. Although no person can always predict the future with perfect accuracy, prognostic attempts for various diseases are based on empiric knowledge from the past. That is, how specific patient populations diagnosed with the disease in question fared over times. Of course this depends on the length of time and on the specific outcome of interest. In medicine, length of follow-up extends either from symptom onset or hospital admission to discharge or few months or years later and specific outcomes range from mortality to cognitive or physical status (dependence or independence). In neurocritical care, prognostication takes on a high priority because many diseases are either fatal or lead to substantial disability. Patients, families, and healthcare providers want to know what to expect and these expectations often influence decisions regarding acute care and long-term support. Furthermore, prognostication is often considered in relation to a specific time point, but may be relevant across a continuum of outcomes and time points. Prognosis for early mortality and long-term functional independence may be important for different types of decisions such as whether to continue aggressive neurointensive care or whether arrangements should be made regarding job prospects and family financial planning. The plateau of functional status and the time course and trajectory to get there may be the most important prospect to patients and families. However, most studies assess a formal outcome at an arbitrary snapshot in time such as hospital discharge, 3 months, 6 months, or 1 year. Prognosis assessment can vary between different disease states and between available variables, scores, and scales defined by the literature.

In February 2018, the Arbeitstagung NeuroIntensivMedizin (ANIM) meeting took place in Würzburg, Germany, and involved substantial collaboration between the German Neurocritical Care Society (DGNI) and the Neurocritical Care Society (NCS). Because of the recognized importance of prognostication in neurocritical care, this collaboration included a special joint session focusing on gaps in current prognostication paradigms and models in neurocritical care. The purpose of this session was to provide a forum for presentation and discussion regarding existing formal prediction models across eight different common neurocritical care conditions (aneurysmal subarachnoid hemorrhage, intracerebral hemorrhage, acute ischemic stroke, traumatic brain injury, traumatic spinal cord injury, status epilepticus, Guillain–Barré Syndrome (GBS) and global cerebral ischemia from cardiac arrest). Members of DGNI and NCS who were recognized as experts in prognostication or clinical care of these conditions (one expert per condition) were selected by the session organizing committee. They were instructed to undertake a general literature search regarding prognostication models for their assigned condition, to present an oral 15 min-summary, and to moderate and integrate feedback from the audience into a brief written summary. Formal defined literature searches or systematic reviews with utilization of a librarian were not undertaken nor was there specific grading of the literature using an established methodological approach. Rather, the intent was to provide an overview for discussion in order to identify gaps in current prognostication models as well as themes that might cross over between different conditions. Recognizing that prognostication paradigms involve broader aspects than just prognostication models, this focus was chosen specifically in order to frame the gap analysis and conform to available program time. This report involves a summary of the individual presentations which were synthesized into this single document by the session moderators. It is hoped that this gap analysis can provide a framework for DGNI and NCS members as well as the neurocritical care community worldwide to improve the study of prognostication tools and methods and guidelines for their use.

## Subarachnoid Hemorrhage

### Outcome Predictors and Prognostic Models

The long-term outcome after aneurysmal subarachnoid hemorrhage (SAH) has improved over the last decades [1–4]. The original grading scales of clinical severity, Hunt and Hess scale [5], and World Federations of Neurosurgical Societies (WFNS) scale [6], are still the most widely used and remain the most important predictors of long-term poor functional outcome and mortality [7, 8]. In comparative research, there was no difference in the precision of outcome prediction between the Hunt and Hess grade and the WFNS using the modified Rankin scale (mRS) and the Glasgow Outcome scale (GOS) at discharge, 6 months and 12 months [9–11]. However, in the WFNS scale there was substantial overlap between grade II and III, III and IV with similar outcomes for the assigned grades [9, 12].

The original scales, Hunt and Hess and WFNS, were modified to enable more precise and reliable distinction between the grades. Most of them are based on the Glasgow Coma scale (GCS) [13]. The modified WFNS scale was better able to distinguish between grades I, II, and III as well as IV and V when predicting the mean GOS and mRS at 90 days [14]. Another GCS-based scale (“Prognosis on Admission of Aneurysmal Subarachnoid Hemorrhage-PAASH scale”) clearly distinguished 6-month outcome based on the GOS and mRS [15, 16]. The Revised GCS-based scale on four significant breakpoints of the admission GCS predicted long-term outcome (mRS at 3 and 12 months) better in poor grade patients compared to GCS, WFNS, and Hunt–Hess scales [17]. The Full Outline of UnResponsiveness (FOUR) score with four scoring items: eye opening, eye and eyelid movements (E), motor examination (M), brain stem reflexes (B), and respiratory patterns (R) were designed for more detailed assessment of the level of consciousness [18]. The total, eye, motor, and respiratory FOUR scores obtained on day 0 and 7 after SAH were associated with mortality and functional outcome (mRS and GOS) at 1 and 6 months [19]. This score obtained on day 14 was also associated with functional outcome (GOS) at 6 months [19]. The SAH Physiologic Derangement score was designed to identify potentially reversible disorders during the acute phase of SAH. This score was found to be a better predictor of death and severe disability (mRS) at 3 months after SAH [20].

Few prognostic models were developed to predict long-term outcome after SAH. Whereas the SAH score is based on patient’s age, admission GCS, and comorbidities [21]; the HAIR score [11, 22] composed of Hunt–Hess score, age, the presence of intraventricular hemorrhage, and rebleeding within 24 h; and the ABC score including GCS, troponin I, and protein S-100ß [23] obtained on admission, focus on prediction of in-hospital, discharge, and long-term mortality. The two more recently developed models, the Functional Recovery Expected after Subarachnoid Hemorrhage (FRESH) score and the Subarachnoid Hemorrhage International Trialists (SAHIT) score aim at prediction of long-term functional outcome and are considered to be the most comprehensive [24–26].

The FRESH score (Table 1) is composed of Hunt–Hess grade, APACHE II physiologic subscore on admission, age, and rebleeding within 48 h to add up to 9 points and prognosticates functional outcome (mRS) at 12 months after SAH. The score was developed based on 1526 SAH patients with exclusion of the patients in whom care was withdrawn (area under the curve [AUC] 89.8%). It was validated in a different cohort of 413 patients (AUC 73.2%). Additional scores for prognostication of cognitive outcome and long-term quality of life at 12 months have been developed, such as the FRESH-cog score and the FRESH-Quol score. Both models were developed in a single-center cohort and have not been externally validated [26].

**Table 1:** FRESH score [26]

FRESH function		
Age	≤ 70	> 70
Hunt–Hess scale	I–V	
Apache II physiologic score	Middle arterial pressure	Heartrate
	Respiratory rate	Temperature
	White blood cell count	Hematocrit
	Sodium	Potassium
	Aa gradient (if FiO_2_ ≥ 50%) or paO_2_	pH or HCO_3_
	Creatinine	
Rebleeding within 48 h	Yes	No
FRESH-Cog	Years of education up to 24 years	
FRESH-Quol	Premorbid glasgow outcome scale	

*FRESH-Cog* functional recovery expected after subarachnoid hemorrhage–cognition at one year after SAH, *FRESH-Quol* functional recovery expected after subarachnoid hemorrhage–quality of life at one year after SAH

The SAHIT score was derived from pooled data of 10,936 patients from several randomized clinical trials, prospective observational studies, and hospital registries and externally validated in 3355 and 338 patients. Outcome was assessed by GOS at 3 months. The final model encompasses age, hypertension, WFNS for the core model; aneurysm size and location as well as Fisher grade for the neuroradiology model; and treatment modality for the full model. The addition of these variables to the core model increased the AUC slightly [24, 25].

### Summary and Gaps

Severity scales such as the original or modified Hunt–Hess scale, WFNS score, and GCS are still most widely used scores to approximate long-term prognosis in daily clinical practice and clinical trials. Most of these scores were developed in cohorts of < 500 SAH patients with single-center or multicenter prospective or single-center retrospective study designs.

Newer prognostic models such as FRESH score and SAHIT score offer more precise long-term functional outcome prediction and are externally validated. Cognitive and psychological outcome measures as well as quality of life are the most disabling in the SAH population and are only accounted for in FRESH-cog and FRESH-Quol scores which have not been not externally validated.

Several gaps in prognostication in SAH exist: the individual patient status prior to the onset of SAH is lacking from most models, except for the FRESH-Quol score. The development of the prediction models is usually based on retrospective analyses of a large patient population, and patients with advanced directives against aggressive measures as well as mortality associated with withdrawal of life support are usually not excluded. All prediction scores are mainly based on variables and parameters obtained on admission, but in-hospital complications are not considered. The optimal time point for assessment of predictors of outcome is unknown. There is only one, thus far unvalidated model, which includes cognitive and psychological outcomes including quality of life, FRESH-cog, and FRESH-quol. Most clinical trials in SAH do not measure these long-term outcomes.

## Intracerebral Hemorrhage

### Outcome Predictors and Prognostic Models

There are more than 20 published prognostic scores for intracerebral hemorrhage (ICH), the majority of which were developed using data from single-center cohorts [27–29]. The ICH score (i.e., “original ICH score,” or oICH score) [30] is the score that has been validated in the largest number of independent patient cohorts [31]. In its initial publication in 2001, the ICH score was associated with 30-day mortality [30]; in 2009, it was validated for functional outcomes out to 12 months [32]. Notable alternatives to the oICH include the ICH-Grading scale (ICH-GS) [33], which considers supratentorial versus infratentorial ICH location; the modified ICH score [34] and Essen ICH score [35], which incorporate clinical examination findings via the National Institutes of Health Stroke scale (NIHSS), as opposed to GCS; the ICH-Functional Outcome score (ICH-FOS) [36], which incorporates both the GCS and NIHSS; the modified Emergency Department ICH (EDICH) score [37, 38], which incorporates a patient’s initial International Normalized Ratio (INR) value; and the Functional Outcome in Patients with Intracerebral Hemorrhage (FUNC) score [39] and Maximally treated ICH (Max-ICH) score [40], which were developed in part by using cohorts in which patients receiving early limitations of care were removed, in an effort to minimize self-fulfilling prophecy effects. Whereas the modified ICH score, the ICH-FOS, the Essen ICH score, the ICH-GS, and the Max-ICH score are associated with in-hospital mortality and long-term poor functional outcome (mRS 3–6) up to 12 months. The modified EDICH score is associated with 48 h and in-hospital mortality, neurological deterioration at 48 h, and poor functional outcome at discharge (mRS > 3) [37, 38]. The FUNC score was designed to predict functional independence in surviving patients [39]. Few of these modifications of the original ICH score were externally validated.

Head-to-head comparisons of ICH prognostic scores over the past decade have compared different scores and different outcomes, a fact making determination of the most robust model a difficult task.

A comparison of eight grading scales in 2011 among 67 ICH patients in New York suggested that the Essen ICH score had the most outstanding discrimination for predicting in-hospital mortality, 3-month mortality, and 3-month functional outcome, although overall differences among the tested scores were minimal [41]. A 2013 study among 501 patients in Texas concluded that the FUNC score and ICH-GS had better discrimination for 3-month mortality and functional outcome than the oICH score [42].

A 2015 meta-analysis conducted in the UK compared 12 ICH-GS and concluded that the oICH score and the ICH-GS had the greatest amount of worldwide supporting evidence for use in predicting early mortality [28]. However, a retrospective analysis of 2556 patients from the INTERACT2 trial (a large trial of blood pressure reduction for acute ICH) published that same year found that the modified ICH score had better discrimination for poor 90-day outcome compared to the oICH and ICH-GS [43].

Subsequently, a 2016 retrospective analysis of 342 ICH patients in Germany suggested an advantage of both the oICH score and ICH-GS over the FUNC score in predicting 30-day mortality, based on Pearson correlation [44]. A 2018 analysis of 1338 ICH patients in Singapore found the ICH-GS slightly more advantageous than the oICH score for predicting the same outcome [45].

A 2017 study of 170 ICH patients in Italy concluded that the modified EDICH score had better discrimination than the oICH score, the ICH-GS, and the FUNC score with regard to predicting early neurological deterioration, in-hospital mortality, and poor functional outcome [46]. Finally, a 2017 study comparing 19 prognostic scores in 882 Scandinavian ICH patients found that the ICH-FOS outperformed all other scores with respect to predicting 3-month and 12-month mortality, although the NIHSS alone performed just as well as the ICH-FOS in predicting in-hospital death [31].

### Summary and Gaps

It is currently unclear what the day-to-day clinical importance should be ascribed to ICH prognostic scores, regardless of which one a clinician chooses to use. Some have advocated for increased use of prognostic scores to help guide clinicians with goals-of-care decision making [47]; however, to date, available scoring systems have not yet been shown to outperform the early subjective clinical judgment of physician and nurses at academic neuroscience centers with regard to predicting functional outcomes [48]. The authors of this document caution against the use of any ICH prognostic score as the sole means for prognosticating outcome for an individual patient.

Although prognostic scores typically incorporate clinical variables available upon hospital admission, the time window that may truly be best for accurate outcome prediction during an ICH patient’s hospitalization course is currently unclear. As the literature evolves from simply warning against premature withdrawal of care [49], recent studies have suggested that reassessing an ICH patient’s clinical exam over a period of 24 h [50] or even 5 days [51] greatly improves prognostic accuracy. Further studies may explore optimal prognostic time periods, including when on average ICH patients achieve “prognostic stability.” Also, there is increasing appreciation for how patient comorbidities and systemic illnesses associated with ICH patients during intensive care unit hospitalizations (i.e., infections, etc.) may ultimately determine patient outcome [52, 53]. There have been some recent attempts to include physiologic measures, such as the APACHE II score, into ICH prognostication models, with varying success (e.g., the Prognosticating Functional Outcome after ICH scores) [54–56]. While such attempts may make subsequent grading scales more complicated to calculate, there is a possibility that further research in this area may ultimately help with improving prognostic accuracy.

Finally, while the early subjective clinical judgment of clinicians at academic neuroscience centers may correlate better than oICH and FUNC scores with the functional outcomes of ICH patients, it is not clear whether the judgment of non-specialist community clinicians would hold an equal advantage in head-to-head testing. A study of the accuracy of outcome prediction from non-neuro-specialist clinicians who nevertheless care for ICH patients could potentially reveal an expanded role for the judicious use of grading scales and their associated outcome data in clinical practice.

## Ischemic Stroke

### Outcome Predictors and Prognostic Models

Model/score-based prognostication after acute ischemic stroke (AIS) aims to predict mortality, long-term functional outcome or complete recovery in particular [57–60]. Moreover, functional outcome and mortality at hospital discharge or after 90 days may be predicted in correlation to stroke-specific treatment [61, 62]. Mainly based on selected cohorts of AIS patients with heterogeneous neurological deficits and medical treatments, variables of prediction models were assessed in a defined setting and functional outcome was measured at varying time points after stroke [58]. In addition, treatment delays, “the withdrawal of care bias” [63], patient-centered preferences [64], and even the type of hospital may have an impact on treatment decisions [65], which in turn affect outcome after AIS. Therefore, generalizability of available prognostic models/scores is limited [60]. In addition, multi-item models/scores may be regarded to be too complex in daily clinical practice, especially if neuroimaging or information outside clinical routine has to be included [58, 66].

The most relevant ethical question is whether there is a threshold of acceptable accuracy of a prognostic model/score, especially regarding decision making to withhold potential life-saving treatments after AIS [60]. While there are more than a hundred studies describing scores to predict functional outcome and/or mortality after AIS [59], these scores should not be an integral part of clinical routine and were not included in AIS guidelines [64, 67].

Every prognostic model has certain strengths and weaknesses. Therefore, a quantitative comparison of the prognostic accuracy of models is a challenge and prone to misinterpretation [57–60]. A recent review focused on multi-item scales to predict outcome at 30 days after stroke. The eight scales (ASTRAL, iSCORE, iSCORE-r, PLAN, SOAR, modified-SOAR, SPI2, and THRIVE) are based on clinical data on admission, but do not include neuroimaging [60]. External validation using patient-level data from the Virtual International Stroke Trials Archive database demonstrated that scales have different discriminative power. In detail, the *Acute Stroke Registry and Analysis of Lausanne* (ASTRAL) scale had a significantly better prognostic discrimination with regard to mRS and the best prognostic discrimination with regard to the Barthel Index or mortality at 90 days after stroke. Interestingly, external validation mostly resulted in lower prognostic discrimination compared to the baseline publication [59, 60].

Another review focused on AIS prognostic models predicting functional outcome at ≥ 90 days based on clinical and/or brain imaging using magnetic resonance imaging (MRI) or computed tomography (CT) and CT perfusion. Overall, seven scales (DRAGON, MRI-DRAGON, HAT, HIAT, HIAT2, NAV, SAD) including neuroimaging data assessed in the acute phase of AIS or during follow-up were compared to seven scales based on clinical information (ASTRAL, BOAS, iScore, NIHSS, sNIHSS-4, SPAN, THRIVE) [58]. Since no relevant differences regarding the discriminative utility of the analyzed scales were detected, the authors conclude that there is no score “that is the obvious choice for all clinical situations” [58].

Based on 10 cohort studies including elderly patients with moderate AIS severity, 23 prognostic models for complete recovery after AIS were analyzed regarding discriminative power and calibration in another recent systematic review [57]. Methodological quality of these models differed. Compared to a model using the NIHSS [68] and stroke volume, multi-predictor models including stroke severity, stroke volume, pre-stroke disability, cardiovascular risk factors, and use of systemic thrombolysis had a similar predictive value. Focusing on a single score, functional outcome at 90 days after AIS is highly associated with stroke severity measured by the NIHSS on hospital admission [64, 69].

### Summary and Gaps

In addition to the limited generalizability of published prediction models, independent internal and external validation of a prognostic model’s calibration and discrimination is a key issue but not available in the majority of published models [57–60]. Moreover, there is considerable heterogeneity in validation studies for certain scores [59]. Of note, periodical recalibration is needed to reflect the impact of novel therapeutic approaches (like thrombectomy) that affect outcome after stroke.

Despite methodological improvements in recent years [59], even well-validated AIS specific models with good prognostic value should not be exclusively used as basis for clinical decision making, especially not in emergency situations [64, 69]. Based on published prediction models, multimodal solutions to improve prognostication after AIS are needed to reduce the chance of misclassification in the individual stroke patient [59, 60].

## Traumatic Brain Injury

### Outcome Predictors and Prognostic Models

This summary focuses on moderate-severe traumatic brain injury (TBI) (post-resuscitation GCS ≤ 12) as patients with this severity are commonly admitted to neurocritical care units. While summarized under a single disease entity, TBI is the most heterogeneous of all acute brain injuries in mechanism, pathology, severity and prognosis [70]. Furthermore, before the common data elements [71, 72] were published with the goal to standardize data collection in TBI-related research studies, the high heterogeneity of variables and outcomes measured in TBI studies made outcome prediction modeling challenging.

For blunt TBI, two major outcome prediction models were published in 2008: the International Mission for Prognosis and Analysis of Clinical Trials in TBI (IMPACT)-model [73] and the Corticosteroid Randomization after Significant Head Injury (CRASH)-model [74]. The IMPACT-model was derived from a large TBI cohort (*n* = 8509) including 11 studies (eight randomized controlled trials and three observational studies) with GCS ≤ 12 and complete 6-month GOS [73]. The model’s outcomes were 6-month mortality and unfavorable outcome (GOS 1–3). Three sub-models were created: “core model,” including age, pupillary reactivity, and motor GCS; “core + CT model,” which additionally included presence of hypoxia (O_2_ saturation < 90%) or hypotension (systolic blood pressure < 90 mm Hg) in the field or emergency department and Marshall CT classification; and “core + CT + lab” model, which additionally included hemoglobin and glucose levels on admission. The “core” and “core + CT” model were externally validated in the CRASH-trial cohort, but the “core + CT + lab” model was not due to lack of laboratory values from the CRASH-trial. For all three models, the discrimination (AUC-ROC) was > 0.8. Calibration, as measured by the Hosmer–Lemeshow test, was adequate (*p* value > 0.05). However, the cumulative *R*^2^ of the full model is 0.35, which means that the IMPACT-model only explains about 1/3 of the outcome variability. The IMPACT-model is the most widely validated TBI prediction model, including its validation in more contemporary cohorts, such as the SYNAPSE-trial dataset [75]. The IMPACT-website (www.tbi-impact.org) contains an online IMPACT-model calculator [76]. Disadvantages of this calculator are the display of outcome in bar graphs instead of IconArrays [77], which are the preferred method of graphical risk communication [78]. A second disadvantage includes the lack of confidence intervals for the estimated outcomes.

The CRASH model was derived in 10,008 patients enrolled in the worldwide CRASH-TBI-trial with GCS ≤ 14; therefore, the CRASH model is not limited to moderate-severe TBI [74]. The model was externally validated in the IMPACT-cohort (*n* = 8509) and predicts 14-day mortality and 6-month unfavorable outcome (GOS 1–3). The CRASH model has the unique feature of differentiating outcome by high-income (*n* = 2482) and low-income countries (*n* = 7526). Compared to high-income countries, TBI in low-income countries were younger, more likely male, recruited later, had less severe TBI (higher GCS, better pupillary reactivity), more often had an abnormal head CT, had higher mortality at 14 days (OR 1.94, 95% CI 1.64–2.3), but there was no difference in unfavorable outcome at 6 months [74]. The discrimination (AUC-ROC > 0.8) was excellent. The calibration was adequate, except for the low-income country models for both outcomes, when including head CT-data (Hosmer–Lemeshow *p* value < 0.05). The CRASH model prediction can be accessed via an online calculator [79].

For penetrating TBI (pTBI), only one prediction score has been published: the predicting survival after acute civilian penetrating brain injuries (SPIN) score [80]. This score was derived from 413 patients retrospectively retrieved from two US Level-1 trauma centers, which poses the largest contemporary cohort of pTBI. The variables included were motor GCS, pupillary reactivity, self-inflicted injury, transfer from other hospital, sex, injury severity score and admission INR. The AUC-ROC was outstanding (0.97); calibration was not reported. While the original SPIN-score publication did not include an external validation, in the meantime the authors have conducted an external validation in a cohort of 362 patients from three US Level-1 trauma centers [81]. In this external validation, both discrimination and calibration were excellent (AUC-ROC 0.88; Hosmer–Lemeshow *p* value > 0.05).

### Summary and Gaps

Many gaps remain in the outcome prediction after blunt and penetrating moderate-severe TBI. One of the major gaps arises from the fact that all aforementioned prediction models include only admission variables, disregarding the commonly long hospital course. This is mostly due to the lack of standardized hospital course data collection in the studies from which these models were derived. Additional variables which may improve the existing prediction models could include concurring in-hospital complications, and the trajectory of improvement or worsening after admission. The second gap, which, if closed, would have the largest impact on patient outcomes, is the standardization and improvement of the risk/outcome communication to patient families [32, 82–84]. This may significantly reduce the influence of anecdotal reasoning and bias by healthcare providers or families on patients’ healthcare decisions [85–87].

## Traumatic Spinal Cord Injury

### Outcome Predictors and Prognostic Models

The available literature of the past decade was reviewed on prognostic scores and models for outcome prediction after traumatic spinal cord injury (SCI). The first clinical prediction rule for ambulation outcomes after 12 months was derived from a longitudinal cohort study (European Multicenter Study on Human Spinal Cord Injury) conducted at 19 centers over a 7-year period. Predictor variables were retrieved from standardized neurological examinations within 15 days of injury as defined by the International Standards for Neurological Classification of Spinal Cord Injury in addition to age [88].

A simplified 3-variable model of this prediction rule was proposed and tested on the basis of acute phase (< 15 days) and outcome (> 12 months) data from the Canadian Rick Hansen Spinal Cord Injury Registry [89]. Demonstrating similar accuracy, it could potentially enhance clinical utility.

Another clinical prediction model for long-term outcome (at 1 year) after traumatic SCI was based on two prospective datasets from the North American Clinical Trials Network for SCI and the Surgical Timing in Acute Spinal Cord Injury Study [90]. It included both clinical and imaging variables obtained in the acute setting (i.e., within 72 h of SCI). Data from the US-multicenter Spinal Cord Injury Model Systems database were used to compare mathematical models for accurately predicting different levels of independence at 12 months following SCI, ambulation status, as well as non-ambulation outcomes from rehabilitation admission examinations (i.e., within 9 weeks of SCI) [88, 91]. All models were based on logistic regression analyses and one employed artificial neural networks in comparison [91]. Classifications of injury severity typically utilize the ASIA impairment scale obtained either on admission or within 72 h. Outcome assessment at one year included ambulation ability (self-reported or examined) and composite functional assessments such as the functional independence measure and/or spinal cord independence measure [92].

### Summary and Gaps

In the domain of traumatic SCI, prognostic prediction models gain clinical significance through their direct impact on decision making and counseling of patients and their relatives. Currently, prognostication of outcomes in SCI is often based on pragmatic single-factor designs utilizing for example the clinical examination and impairment grading in the acute phase, although later time points (e.g., at rehabilitation admission) might be of equal importance. Novel diagnostic biomarkers such serum proteins are not used, and data from clinical imaging are rarely incorporated. The variation of outcome measures can limit the value of prognostication in the individual case. In the future, multidimensional outcome measures of neurological status, functional performance, and psychosocial well-being could bring the complexity of traumatic SCI and post-injury course into the limelight.

Improved prognostic modeling could fill the gap in order to inform (a) patient and relatives as to the odds for specific outcomes, (b) clinicians for individualized therapy guidance, and (c) clinical and genetic-molecular researchers/statisticians in the development and testing of innovative therapeutic measures.

## Status Epilepticus

### Outcome Predictors and Prognostic Models

Status epilepticus (SE) is characterized by marked heterogeneity in both symptoms and underlying etiologies, making prediction of prognosis particularly challenging. In general, the impact of persisting seizures on outcome diminishes with growing severity of the condition that causes them. Although, in a considerable proportion of cases, prognosis of SE is clearly dominated by an underlying disease, SE can nonetheless have an impact on the outcome either through direct neuronal damage or by being a source of complications [93, 94]. Terminating SE as quickly as possible is, therefore, a well-accepted therapeutic goal in neurocritical care. However, treatment of SE can be hazardous, especially when it is refractory to anticonvulsants and anesthetics have to be applied [95]. By identifying patients with an a priori high chance of good outcome, prognostication scores could help recognize cases with a less favorable risk–benefit ratio.

Prognostication tools providing clinicians with this information should be easily applicable and be based on readily available patient data, particularly as SE calls for timely intervention in most cases. To date, four prediction tools have been created aiming to prognosticate in-hospital mortality of SE based on different sets of prognosticators. The first ever published score for prediction of outcome in SE, the Status Epilepticus Severity score (STESS), meets this requirement, as calculation of the score does not need any additional diagnostics [96]. In several studies, STESS was repeatedly found a reliable predictor of SE outcome. Uncertainty remains, however, regarding the optimal STESS threshold that differentiates SE survivors from non-survivors [97]. Recently, an increase in prognostic accuracy by adding premorbid functional status to STESS was proposed in a modified version of STESS [98]. Leitinger and colleagues introduced another score, putting emphasis on detailed differentiation of SE etiologies with their impact on SE prognosis graded by mortality rates which were taken from previous epidemiological studies (Epidemiology-based Mortality score in SE) [99]. Finally, a group from China recently introduced the END-IT score, based, among others, on imaging findings and information on response to first-line treatment [100]. This score was derived from a cohort of young intensive care unit patients with a high proportion of encephalitis underlying SE. This may limit its applicability to a setting in Europe or North America where patients with SE are mainly elderly suffering from various etiologies.

### Summary and Gaps

Overall, external validation of SE prognosis scores is scarce and results of comparisons of score performances are controversial [101]. In addition to this shortcoming, there are among others two challenges which are not addressed by the current scores. Long-term functional outcome is not reflected by the current scores which mainly focus on in-house mortality and the prediction of development and severity of epilepsy after an episode of SE is not included in current prognostication tools. In summary, the current value of SE outcome scores possibly lies in assisting clinicians to find optimal treatment for their patients in the early stages of a SE episode. Low positive predictive values for in-hospital mortality may argue against early withdrawal of therapy [101].

## Guillain–Barré Syndrome

### Outcome Predictors and Prognostic Models

GBS is characterized by a monophasic course that uniformly shows improvement over time after reaching its nadir. However, it can be acutely fatal from respiratory failure and complications of dysautonomia, and recovery is often slow and sometimes incomplete [102, 103]. Prognostic studies on GBS have focused on two main endpoints: need for mechanical ventilation and recovery of independent ambulation at 6 months.

### Respiratory Failure

The Erasmus GBS Respiratory Insufficiency score (EGRIS) incorporates the pace of progression of weakness, the presence of facial or bulbar weakness, and the severity of the appendicular weakness on admission into a simple tool that has been shown to predict accurately the need for mechanical ventilation within the first week of hospitalization (AUC 0.84 on the derivation cohort of 397 patients and AUC 0.82 on a separate validation cohort of 191 patients) [104]. The results of pulmonary function tests at the bedside (vital capacity < 20 mL/kg, maximal inspiratory pressure worse than − 30 cm H_2_O, maximal expiratory pressure < 40 cm H_2_O, or decrease of any of these parameters by more than 30%) [105], electrophysiological findings (demyelinating features) [106], and high plasma cortisol level [107] have also been shown to be associated with the need for mechanical ventilation, but these associations have not been sufficiently validated in independent cohorts.

### Functional Outcome

Functional outcome as defined by independent ambulation at 6 months can be reliably estimated using the Erasmus GBS outcome score (EGOS, Table 2) at 2 weeks, which was shown to have adequate calibration and very good discriminative ability (AUC 0.85) both on a derivation cohort (*n* = 388) and on a separate validation cohort (*n* = 374) [108]. A modified version of EGOS (mEGOS, Table 3) that can be applied upon hospital admission or at 7 days was also demonstrated to have good calibration and discrimination (AUC 0.75 upon admission and 0.77 at 7 days) [109]. Other chemical (e.g., IgG-1 subclass of anti-GM1 antibodies, hyponatremia from syndrome of inappropriate secretion of anti-diuretic hormone (SIADH)), electrophysiological (severe conduction block in common peroneal nerve, very reduced amplitude of distal compound muscle action potentials (CMAP) or proximal to distal CMAP ratio of the peroneal nerve < 55.6%), or demographic factors (age older than 40 years) may also predict worse functional recovery [102, 106], but have not been incorporated into prediction scores.

**Table 2:** The Erasmus GBS respiratory insufficiency score

Factor	Categories	Score
Days between onset of weakness and hospital admission	> 7 days	0
	4–7 days	1
	≤ 3 days	2
Facial or bulbar weakness at hospital admission	Absent	0
	Present	1
Medical research counsel sum score at hospital admission	60–51	0
	50–41	1
	40–31	2
	30–21	3
	≤ 20	4
EGRIS		0–7

*EGRIS* Erasmus GBS respiratory insufficiency score

**Table 3:** Modified Erasmus GBS outcome score

Factor	Categories	Score
Age at onset (years)	≤ 40	0
	41–60	0.5
	> 60	1
Diarrhea (within previous 4 weeks)	Absent	0
	Present	1
Medical research counsel sum score	51–60	0
	41–50	3
	31–40	6
	0–30	9
EGOS		1–12

*EGOS* Erasmus GBS outcome score

### Summary and Gaps

The EGRIS, which may be combined with pulmonary function test results, can identify GBS patients who will need mechanical ventilation, while the EGOS and mEGOS, which may be complemented by electrophysiological data, can discriminate early those patients with GBS who will remain non-ambulatory at 6 months. These prediction tools are simple, practical, and applicable to all cases of GBS. Yet, there is a lack of information on the main determinants of quality of life and long-term disability (especially beyond independent ambulation) among GBS patients. These two endpoints should be addressed in future prospective research by incorporating scales of quality of life and emotional well-being and extending the follow-up to examine persistent residual deficits and reinsertion into the workplace.

## Cardiac Arrest

### Outcome Predictors and Prognostic Models

The earliest prognostic scores for unresponsive patients following cardiac arrest came from the sentinel work of Levy et al. from Cornell University in 1985 [110]. Their methods were limited by the technologies available at the time, and thus consisted only of the clinical examination findings during the first 2 weeks post-arrest. However, this laid the groundwork for future studies, and several of their findings hold true to the modern day, such as absence of pupillary and corneal reflexes correlating with poor long-term functional outcome and mortality. Few studies have replicated or modernized their methods, but in 2013 a comprehensive evaluation of 200 comatose cardiac arrest patients was published, incorporating the results of ancillary tests, including electrophysiology and neuroimaging [111]. Once again, the findings of absent pupillary or corneal reflexes strongly correlated with poor outcome at 6 months (modified Rankin scale 4–6) with narrow confidence intervals for false positivity; however, previous indicators, such as absent or extensor motor findings, or myoclonic status epilepticus (MSE), now displayed unacceptably high false positive rates. These findings were confirmed by other modern studies.

Four major guidelines have provided prognostic scoring systems: the 2006 American Academy of Neurology (AAN) Guidelines [112], the 2013 Swedish Resuscitation Council Guidelines [113], the 2014 European Society of Intensive Care Medicine (ESICM) Guidelines [114], and the 2015 American Heart Association (AHA) Guidelines [115].

The 2006 AAN Guidelines were based on studies that were largely performed prior to the widespread use of therapeutic hypothermia (TH), which subsequently has become widely utilized. The AAN Guidelines suggested poor outcome defined as death or unconsciousness after one month or unconsciousness or severe disability after 6 months was associated with MSE on day 1, absent N20 responses on somatosensory evoked potential (SSEP) testing on days 1–3, a serum neuron-specific enolase (NSE) level of > 33 ng/ml on day 1–3, or absent pupillary or corneal reflexes, or absent or extensor motor response on day 3 post-arrest. Subsequent studies have described good outcomes, mainly assessed at 6 months after cardiac arrest, even in some patients with MSE and levels of NSE > 33 ng/ml, and suggested that prognostication is best delayed until the influence of hypothermia can be minimized/eliminated.

The Swedish Guidelines were the first to account for the influence of hypothermia, advocating waiting 72 h after completely rewarming before definitive testing for prognosis. These guidelines also separated their recommendations into early and late predictors, both for good and poor functional and cognitive outcome at 6 months, although the basis of the recommendations was often notably based on weak evidence.

The ESICM guidelines also recommended a waiting period in the setting of TH, as well as the exclusion of confounders, particularly residual sedation. Motor response was used as a screening tool for potential poor functional outcome, but not for definitive prognostication. Patients with absent pupillary and corneal reflexes, or bilaterally absent N20 responses on SSEP, were felt “very likely” to have a poor functional outcome assessed at 6 months in most studies considered. Subsequently, two of four findings (MSE ≤ 48 h post-return of spontaneous circulation, “high” NSE levels, non-reactive burst-suppression or status epilepticus on electroencephalography (EEG), or “diffuse” anoxic brain injury on brain CT/MRI) made poor functional outcome “very likely.” They also emphasized the use of multimodal prognostication whenever possible.

Finally, the AHA Guidelines advocate waiting 72 h after return to normothermia, and postulated that neither absent corneal reflexes nor poor motor responses are reliable predictors of poor outcome. MSE was felt still to be reliable in combination with other tests, and EEG findings of non-reactivity, intractable status epilepticus or a persistent burst-suppression pattern were associated with poor functional outcome (mostly at 6 months). SSEP should be performed 24–72 h post-rewarming, and imaging, including CT or MRI could also be helpful, although without any quantitative guidance for the findings. Finally, NSE and S-100B should not be used alone, but “high levels” could support a poor long-term prognosis for functional outcome.

### Summary and Gaps

Neuroprognostication in cardiac arrest has evolved with the introduction of TH, and modern approaches have advocated for the use of longer waiting periods and a multimodal approach. The clinical exam remains central and paramount, but ancillary testing, including electrical, imaging, and chemical, holds promise for future studies and incorporation into guidelines. It is worth noting that outcomes are typically assessed in cardiac arrest using mRS or the Glasgow–Pittsburgh Cerebral Performance Category (CPC) scale at 6 months, and the definition of a poor versus good outcome varies between studies. Typically, a mRS score of 0–3 or a CPC score of 1–2 is considered good outcome. Furthermore, the timing of assessment of outcome is variable, including at discharge, 3 months and 6 months, although a few studies include assessments up to 12 months after cardiac arrest. The guidelines listed above are based on an amalgamation of studies using different methods of assessment of outcome, and at different time points. The two largest gaps in prognostication after cardiac arrest are 1) lack of blinding of the tests being studied, leading to a behavior confirmation effect; and 2) premature withdrawal of life-sustaining therapy, leading to a self-fulfilling prophecy. Only when these gaps are eliminated will we have quality studies on which to base future recommendations.

## Conclusions

Prognostication is inherent in the delivery of neurocritical care. For essentially every patient admitted to neurocritical care, the question arises “how are they going to do.” Prognostication is undertaken by clinicians using a variety of methods which include informal impressions based on experience as well as formal mathematical prediction tools derived from analysis of populations of patients. Common concerns regarding prognostication include accuracy of prognostic models and the information upon which prognosis is assessed, how this information is delivered to patients and families in order to ensure patient-centered shared decision making and limit physician bias, and avoidance of the self-fulfilling prophecy of poor outcome if care is limited in a patient that might otherwise do well. Although the scope of the joint DGNI-NCS prognostication session at the ANIM meeting was purposely limited to prognostic models, all of the above concerns were raised across the conditions discussed, especially regarding conditions such as TBI, ICH, and cardiac arrest in which patients may be at high risk of early death. Additionally, expanded concerns that are often not explicitly addressed regarding long-term prognostication for outcomes of interest such as ambulation, return to work, and chronic pain in spinal cord injury and GBS were raised as important issues for which there is limited information.

Gaps in prognostication were identified for all the conditions presented (Table 4). Most gaps are common to all disease states. However, the gaps listed in Table 4 represent the most salient ones given the available prognostic tools. Two common themes that emerged across several conditions related to the lack of integration of comorbid conditions into most prognostication models and the fact that in-hospital events were uncommonly included in most models. While some models do include simple measures as a pre-event functional status, it was commented that treating clinicians routinely consider medical comorbidities in their assessment of prognosis and this may explain why some studies have found clinician assessment of prognosis superior to the performance of a specific model. Additionally, while some models include simple information about treatments (e.g., was an aneurysm coiled or clipped; was a traumatic intracranial lesion evacuated), none included robust information about response to treatment, clinical course overtime, and expanded physiology. This information about in-hospital events was also considered to include trajectory. The idea that the pace of a patient’s early improvement or lack thereof as identified by clinical reassessment is an important consideration resonated with numerous presenters and audience participants. However, clinical reassessment over time is not incorporated into essentially any prognostic model presented. In addition to known concerns about the self-fulfilling prophecy, the absence of information about comorbidities, in-hospital care, and trajectory are major gaps identified in this analysis and should be addressed in future studies. Additionally, prognosis for favorable quality of life and the potential discordance between this and formally assessed functional outcome remain significant gaps across all conditions described. Finally, the recognition of post-intensive care stress disorder in patients and even families has not been a focus of prognostication. As more attention is paid to shared decision making, quality of life, and well-being, this gap deserves to be addressed.

**Table 4:** The major prognostication gaps for neurocritical care disease states

Neurocritical care condition	Gap
Aneurysmal subarachnoid hemorrhage	Pre-SAH status not considered in most models In-hospital treatments and complications not included in most models Optimal time point for assessment of predictors
Intracerebral hemorrhage	Models assess parameters at hospital admission, but later assessment may be more accurate Physiology, comorbidities, and in-hospital care not included in prognostic models
Acute ischemic stroke	Lack of external validation of most models New treatments not incorporated into most models
Traumatic brain injury	Existing models incorporate only admission parameters and not in-hospital treatments or complications Lack of standardization of how prognostic information is delivered to patients and families allows for physician bias Optimal time point for assessment of predictors
Traumatic spinal cord injury	Variation in outcome measures leads to limited information for function-specific prognostication Lack of biomarkers, genetics, advanced imaging in existing models
Status epilepticus	Lack of validation of models Long-term functional outcome not assessed in most models
Guillain–Barré Syndrome	Lack of prognostic information on quality of life and long-term disability (besides ambulation)
Cardiac arrest	Lack of blinding in clinical care of diagnostic tests and examination findings evaluated as outcome predictors Self-fulfilling prophecy of poor outcome due to withdrawal of support

*SAH* subarachnoid hemorrhage

In summary, we present an overview of numerous existing prognostic models across eight common neurocritical care conditions. This gap analysis identified several specific targetable issues both within individual diseases and across the group of conditions discussed. The format of initial expert presentation with extensive audience discussion was felt to be particular effective for this initial cross-society collaboration regarding this topic. The gaps highlighted in the summary for each condition will be addressed in a forthcoming formal neuroprognostication guideline based on a literature search by a medical librarian, systematic risk-of-bias assessment with standardized instruments, and application of GRADE methodology. Furthermore, some of the gaps identified in this report need to be addressed by expanded studies of outcome predictors and prognostic modeling.
